# Contact dermatitis-like cutaneous leishmaniasis in a Libyan HIV patient

**DOI:** 10.1186/1756-3305-7-401

**Published:** 2014-08-29

**Authors:** Hamida Al-Dwibe, Aisha Gashout, Abdu-Maged Morogum, Said El-Zubi, Ahmad Amro

**Affiliations:** Faculty of Medicine, Dermatology Department, University of Tripoli, Tripoli, Libya; Faculty of Medical Technology-Pathology Department, University of Tripoli, Tripoli, Libya; Faculty of Pharmacy, Alquds University, Main Campus, P.O. Box 5100, Abu Dis Jerusalem, Palestine

**Keywords:** Cutaneous leishmaniasis, Contact dermatitis, HIV, Sodium stiboglyconate (Pentostam), Libya

## Abstract

**Background:**

Cutaneous leishmaniasis (CL) is one of the common tropical protozoal diseases caused by various *Leishmania* species, and transmitted by the sand-fly vectors, *Phlebotomus* and *Lutzomyia* species. Herein, we report for the first time a case of CL that presented as large eczematous plaques occurring on the dorsi of both feet in a Libyan drug addicted, alcoholic patient with HIV infection.

**Findings:**

A 34 year-old HIV-positive, alcoholic, drug addicted Libyan male presented to us with a history of a non-itchy skin lesions on the dorsi of both feet of 5-weeks duration. Systemic and topical antibiotics were given without improvement. Diagnosis of this patient was confirmed by observation of *Leishmania* amastigote bodies in stained slit-skin smear skin biopsy. After parenteral administration of sodium stiboglyconate (Pentostam) (20 mg/kg/day) for 28 days the lesions did not show any marked improvement. Concurrently, combination therapy of oral rifampicin (600 mg/day) and isoniazide (300 mg/day) was given for 8 weeks. Complete healing of lesions was achieved after this treatment and skin-slit smears turned negative.

**Conclusions:**

Localized cutaneous leishmaniasis should be remembered in deferential diagnosis of unresponsive contact dermatitis especially for HIV-positive patients in CL endemic areas.

This patient was not responding to Pentostam therapy, which is not very common in Libya. Interestingly, combination of oral rifampicin (600 mg/day) and isoniazide (300 mg/day) can be a successful alternative therapy.

## Findings

A 34 year-old HIV-positive, alcoholic, drug addicted Libyan male presented to us with a history of non itchy skin lesions on the dorsi of both feet of 5-weeks duration. The lesions started as small non itchy papules which gradually increased in size and then ulcerated with offensive odour. The patient applied warm onion to treat the lesions, which turned painful and for that he sought medical advice. Systemic and topical antibiotics were given without improvement. The patient denied history of trauma, insect bite, unbalanced diabetes, or allergic diseases and no family history of the same illness.

**Figure 1:**
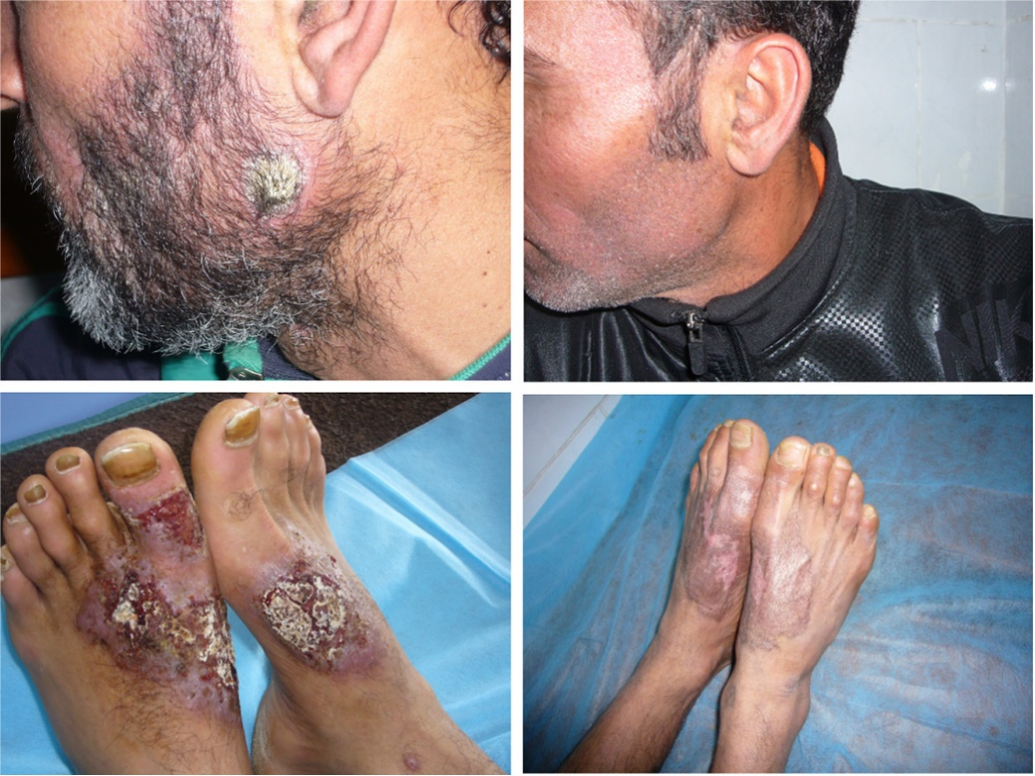
**Painless crusted nodule over left cheek at angle of mandible and bilateral asymmetrical large well-defined erythematous ulcerated plaques with crusts over dorsi of feet (left images).** Complete healing after two months treatment by Rifampicin and Isoniazide (right images).

**Figure 2:**
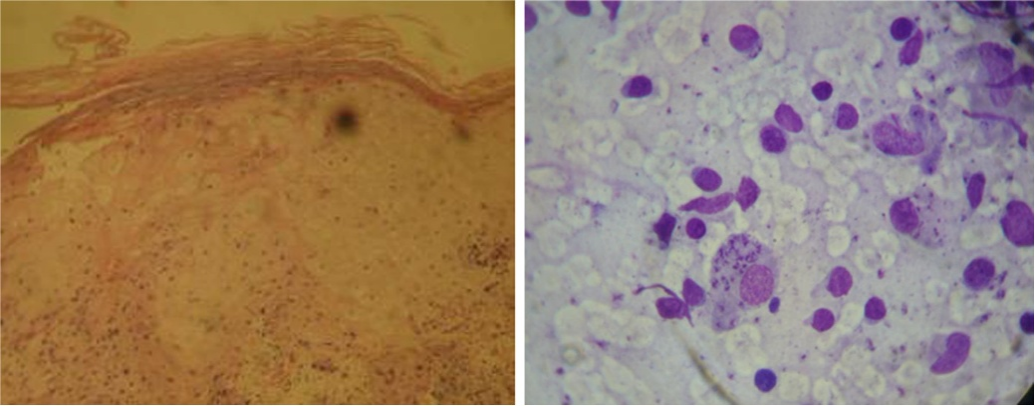
**Intra-and extra cellular** ***Leishmania*** **parasites as shown in Giemsa-stained slit-skin smear (Left).** Histopathological findings of lesions obtained from dorsal surface of right foot (right).

On examination, there were bilateral asymmetrical large well-defined erythematous ulcerated plaques with pus discharge and crusts involving the dorsal surface of both feet and left big toe. Yellowish brown discoloration of the surrounding skin and nails was noticed (Figure 1). In addition, we noticed the presence of painless crusted nodules over the anterior aspect of the right ankle joint and left cheek (Figure 1). No palpable subcutaneous nodules or regional lymph nodes were detected. The patient had a coated tongue and seborrheic dermatitis over his scalp, face and chest. KOH for *Candida albicans* examination was positive from tongue and negative from nails. Routine laboratory tests showed low leukocyte count (3.5 × 103/ul), lymphopenia (16.6%), low platelets (132 × 103/ul), CD-4 T-cell count was lower than (15/ul), and positive serology for HIV and hepatitis C. Other routine investigations were normal including chest X-ray and ECG. Histopathological features revealed focal hyperkeratosis and focal parakeratosis. Stratum spongiosum showed intercellular oedema, local necrosis, and moderate acanthosis. Dermis showed diffuse and nodular mixed cellular infiltration with many extravasated RBCs (Figure 2). No characteristic tubercular granulomas were seen. The clinical picture was consistent with acute contact dermatitis with secondary bacterial infection. However, a 2 mm punch biopsy and slit-skin smear was obtained from all lesions. Smears were fixed and stained with Giemsa’s stain. *Leishmania* parasites were heavily seen inside and outside infected macrophages (Figure 2). Molecular identifications of the causative *Leishmania* species by amplifying the internal transcribed spacer1 (ITS1-PCR) [1] failed due to PCR inhibition problems. After parenteral administration of sodium stiboglyconate (Pentostam) (20 mg/kg/day) for 28 days the lesions did not show any marked improvement, and skin-slit smears were positive for *Leishmania* parasites after this period. The patient was referred to the infectious department for antiretroviral therapy and further HIV care. Concurrently, combination therapy of oral Rifampicin (600 mg/day) and Isoniazide (300 mg/day) was given for 8 weeks. Complete healing of lesions was achieved after this treatment and skin-slit smears turned negative (Figure 1).

Cutaneous leishmaniasis (CL) is one of the common tropical protozoal diseases caused by various *Leishmania* species, and transmitted by the sand-fly vector of *Phlebotomus* and *Lutzomyia* species. About 1.5 million new cases are reported each year, and over 350 million people live in areas of active parasite transmission [2–5]. CL is a major public health problem in Libya caused by *L. major* and *L. tropica* [6]. *Leishmania* species can cause a wide spectrum of cutaneous lesions such as localized cutaneous, muco-cutaneous, diffuse cutaneous, or post-kala-azar dermal leishmaniasis [7, 8]. In HIV-positive patients: asymptomatic and mucosal leishmaniasis has been reported in addition to other usual types [9]. However, HIV-positive intravenous drug users (IVDUs) can be infected with *Leishmania* by sharing of contaminated syringes and needles [9]. Clinical variation of leishmaniasis –HIV co-infection is determined by parasite species and host cell mediated immunity (CMI) response [2]. Recently, clinical variants of CL with or without HIV co-infection have been reported such as; sporotrichoid, psoriasiform, warty, erysipeloid, impetigo like, cold cellulitis, zosteriform, acneiform, and eczematoid variant [10–12]. The incidence of eczematoid variant of localized CL seems not to be very common and reported to be 2.3% [11].

Herein, we report for the first time a case of Pentostam unresponsive CL that presented as large eczematous plaques occurring on the dorsi of both feet in a Libyan drug addicted patient with HIV infection. Localized CL typically presented as papules, nodules, plaques, ulcerated or crusted nodules. A contact dermatitis like morphology of localized CL lesions is unusual. Diagnosis of CL was confirmed by observation of parasites in stained slit-skin smears. This patient was unresponsive to Pentostam, which is not very common in Libya. Combination therapy of oral rifampicin and isoniazide completely cured the lesions. This combination therapy is used in Libya to treat cutaneous tuberculosis and to treat CL cases unresponsive to parenteral administration of Pentostam [6]. A previous study by Peters *et al.* [13] descried a striking remission of CL in a Brazilian patient by using this combination. Small scale studies on oral rifampicin treatment of CL have shown a healing rate of 73.9%, 75% and 83.3% [14–16] respectively. However, no clinical trial was carried out to approve combination therapy of rifampicin and isoniazide in treatment of CL [13].

The patient denied insect bites though he came from Gabel Garby where CL is endemic, possibly the patient was unnoticeably bitten during drug and alcohol intake.

According to ethical approval of this study, the patient agreed to publish his photos and case history anonymously. Written informed consent was obtained from the patient. The study was revised and approved by (Research Ethics committee, University of Tripoli, Libya).

## Conclusion

The learning points from this short report is that localized cutaneous leishmaniasis should be considered in differential diagnosis of unresponsive contact dermatitis especially for HIV-positive patients in CL endemic areas. A combination of oral rifampicin (600 mg/day) and isoniazide (300 mg/day) can be successful alternative therapy for Pentostam unresponcive CL patients in Libya. However, this combination therapy has to be assessed at a larger scale.
